# Supplementary material for: Intranasal infection and contact transmission of Zika virus in guinea pigs
Source: Nat Commun. 2017 Nov 21;8:1648. doi: 10.1038/s41467-017-01923-4 (PMC5698318; doi:10.1038/s41467-017-01923-4)
Supplement: Supplementary file 1 — Supplementary Information [file 41467_2017_1923_MOESM1_ESM.pdf]

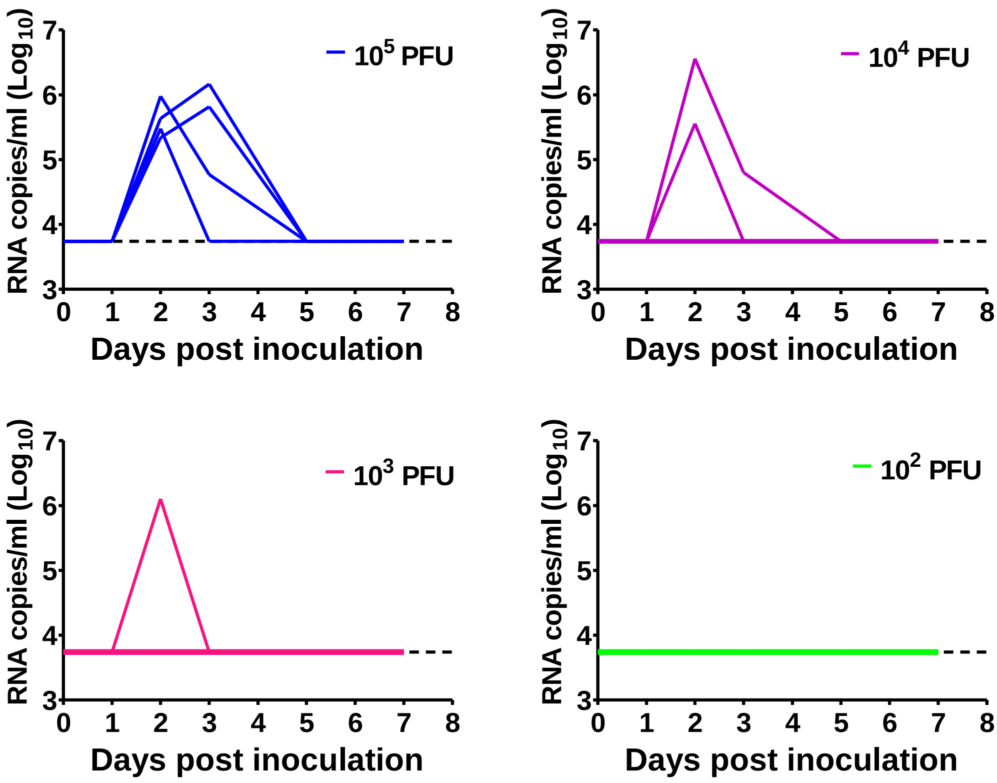

Supplementary Figure 1. Determination of median infectious dose (MID_{50}) of ZIKV in guinea pigs by the s.c. route. For the median infectious dose (MID_{50}) studies, 10-fold dilutions of ZIKV GZ01 strain between 10^5 to 10^2 PFU were inoculated to male guinea pigs (Hartley strain) (four animals per dilution) by the s.c. route. The animals were monitored for survival, weight loss, and clinical signs of disease. Sera of ZIKV-inoculated guinea pigs were collected and analyzed by RT-qPCR for measuring viremia. The MID_{50} of ZIKV in guinea pigs was calculated using the Reed-Muench method.

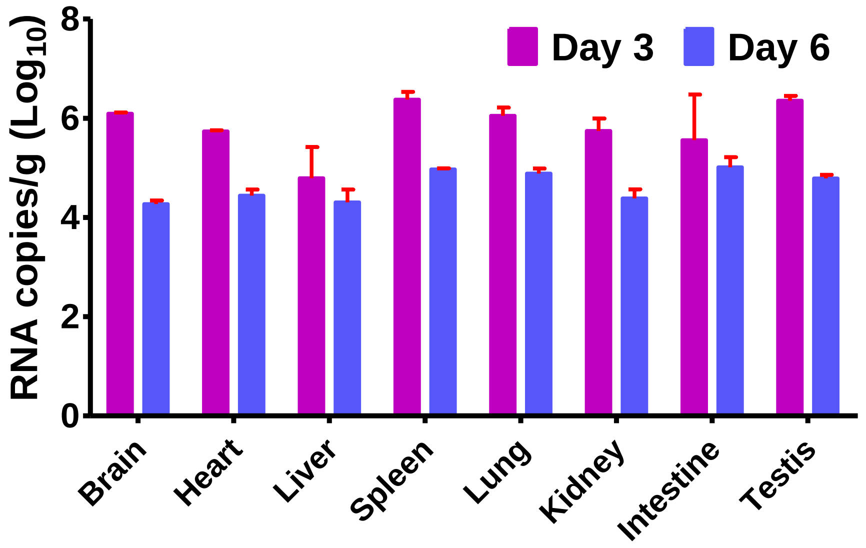

Supplementary Figure 2. Tissue distribution of s.c. inoculated guinea pigs. At 3 and 6 dpi, the various indicated tissues of two anesthetized animals were harvested, weighed, homogenized, and analyzed by RT-qPCR for determining tissue distribution. Viral loads are expressed as RNA copies per gram. Data are presented as mean ± SD.

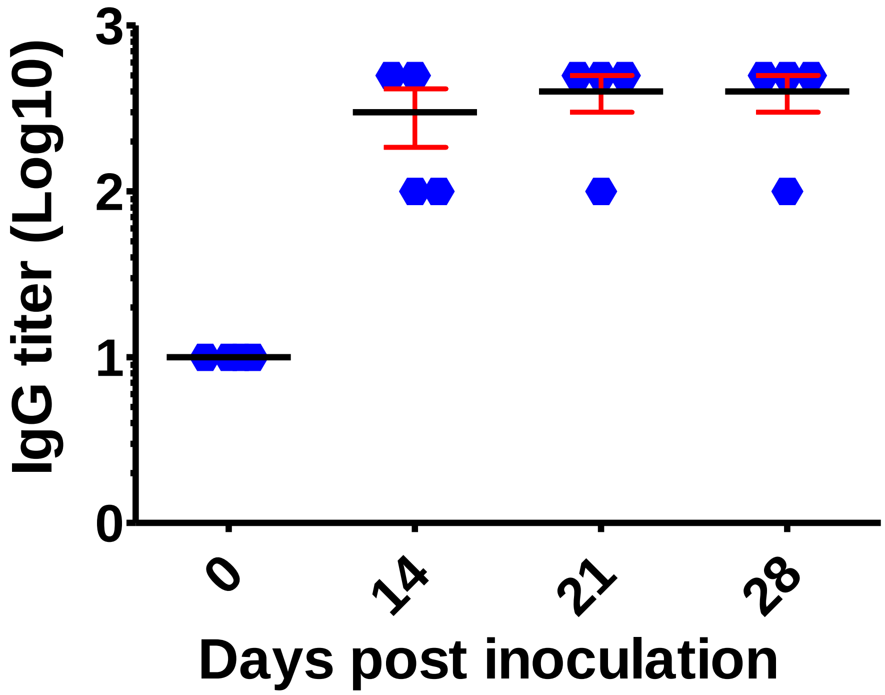

Supplementary Figure 3. ZIKV E-specific IgG antibody response of s.c. inoculated guinea pigs measured by enzyme-linked immunosorbent assay (ELISA). Sera of four inoculated guinea pigs were collected at 0, 14, 21 and 28 dpi. The ZIKV E-specific antibody titers were calculated according to the highest reciprocal dilution of serum to give an OD greater than the sum of the background OD plus 0.01 units. Data are presented as mean ± SD.

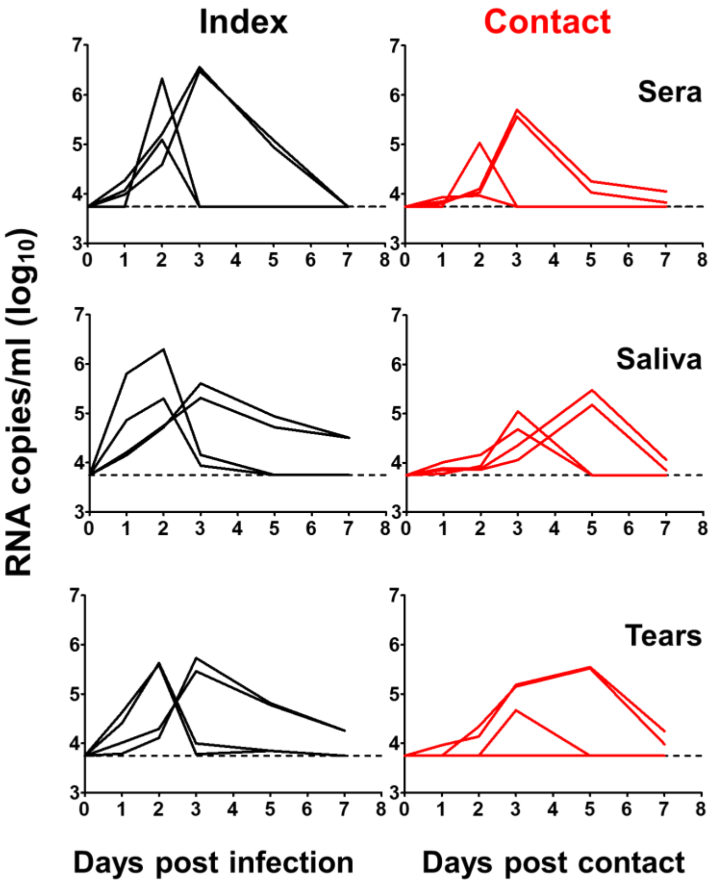

Supplementary Figure 4. Transmission of ZIKV GZ01 strain in guinea pigs. Following s.c. inoculation with 10^5 PFU of ZIKV, each of four male guinea pigs was placed in a cage with one naïve male guinea pig at 3 dpi. Sera, saliva, and tears samples were collected and analyzed followed as above.

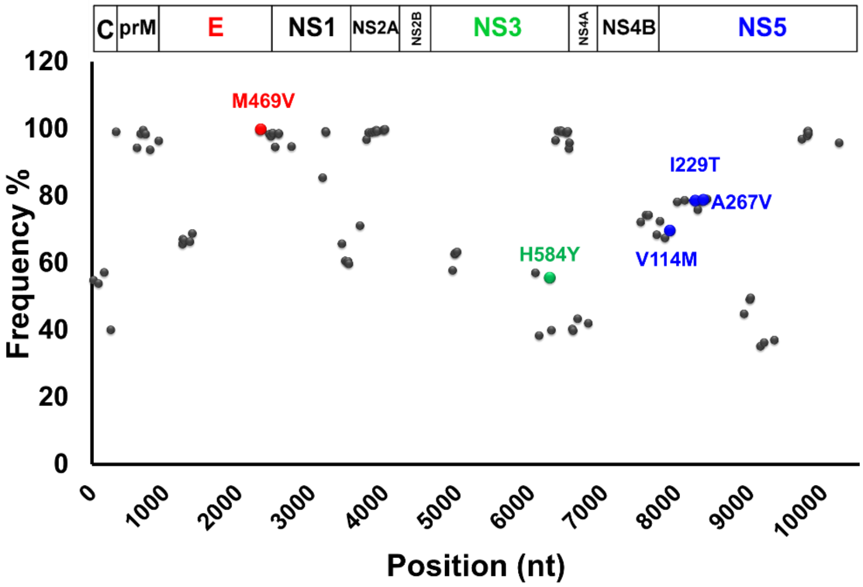

Supplementary Figure 5. Intra-host single nucleotide variants (iSNV) and genetic diversity of ZIKV in parotid glands from guinea pigs at 6 dpi. The amino acid substitution in E, NS3 and NS5 proteins were indicated in red, green and blue, respectively.

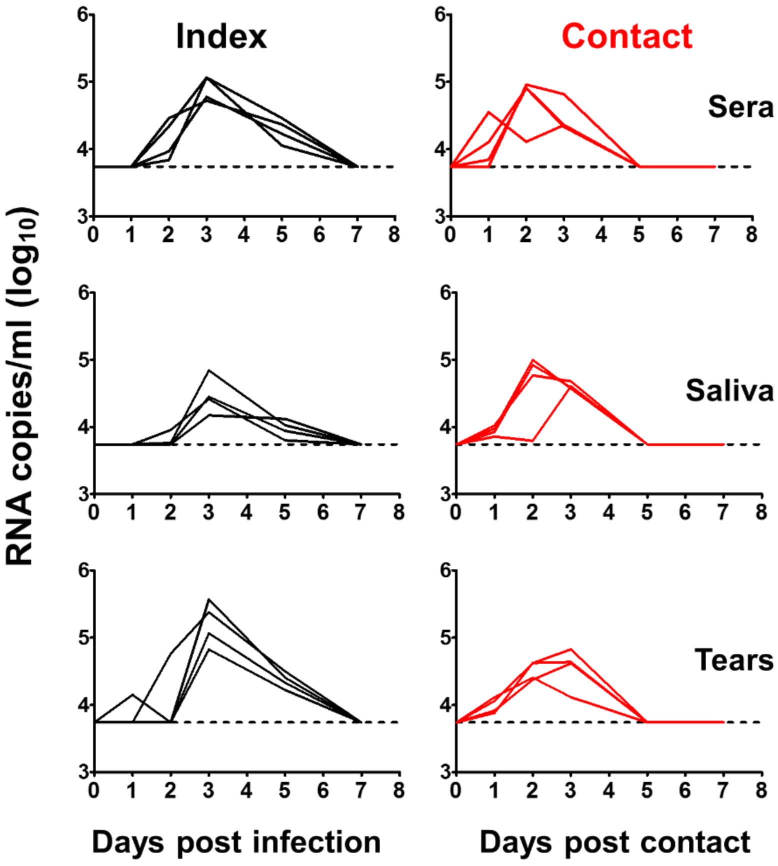

Supplementary Figure 6. Contact transmission of ZIKV strain FSS13025 in guinea pigs. Sera, saliva, tears samples of four inoculated and four contact guinea pigs were collected and analyzed followed as above.

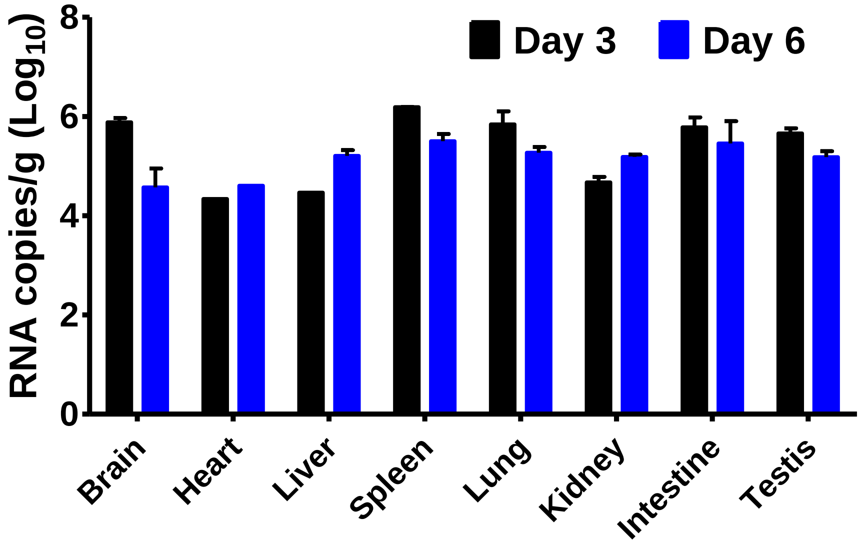

Supplementary Figure 7. Tissue distribution of ZIKV RNAs in the i.n. inoculated guinea pigs. At 3 and 6 dpi, the various indicated tissues of two anesthetized animals were analyzed followed as above. Viral loads are expressed as RNA copies per gram. Data are presented as mean ± SD.

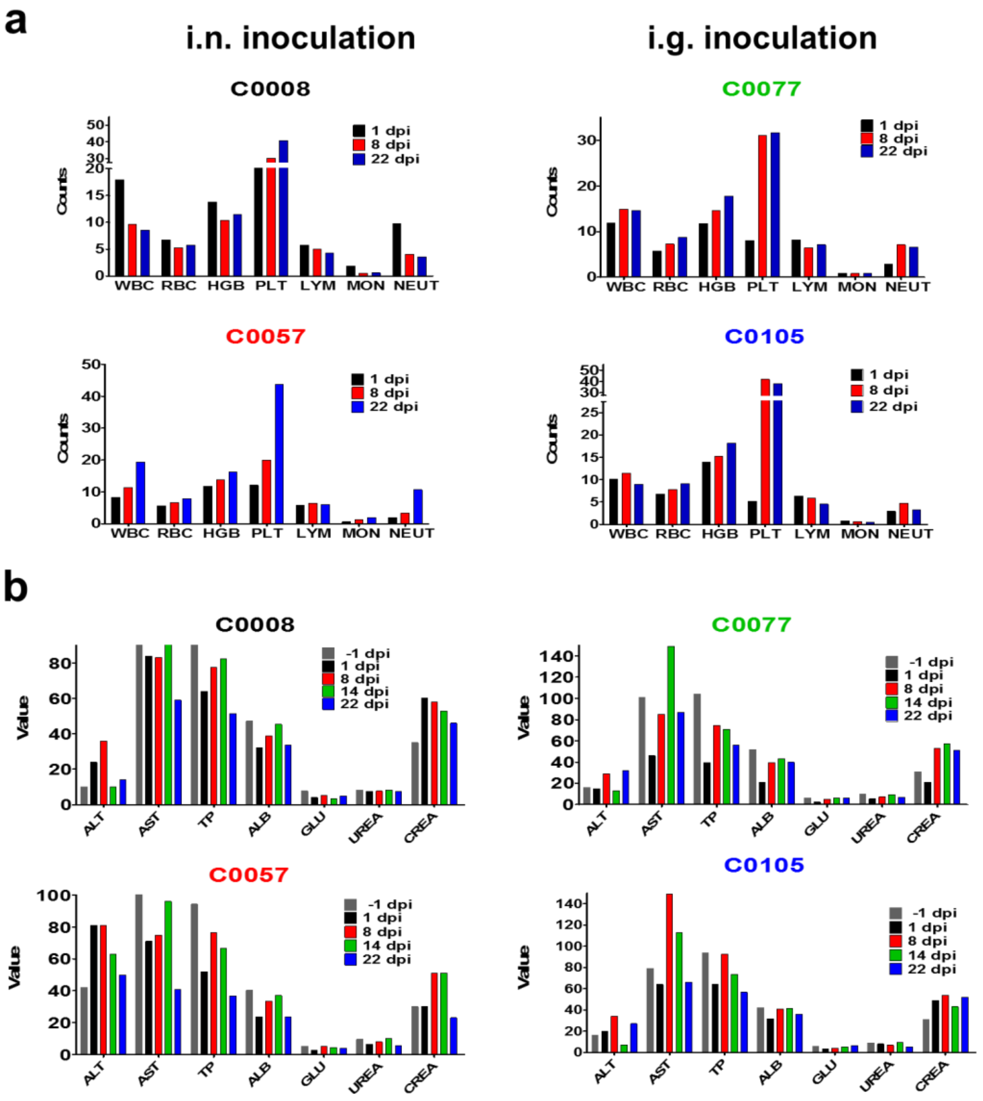

Supplementary Figure 8. Characterization of ZIKV infection in cynomolgus monkeys upon i.n. or i.g. inoculation. Following i.n. or i.g. inoculation with ZIKV GZ01, (a) Complete blood counts (white blood cell (WBC), red blood cell (RBC), hemoglobin (HGB), platelets (PLT), lymphocytes (LYM), monocytes (MON) and neutrophils (NEUT)) were measured at 1, 8 and 22 dpi. (b) Blood chemistries (alanine amino-transferase (ALT), aspartate aminotransferase (AST), total protein (TP), albumin (ALB), glucose GLU, urea (UREA) and creatinine (CREA)) were measured prior to infection and at -1, 1, 8, 14 and 22 dpi.

Supplementary Table 1. ZIKV isolation on C6/36 cells

Sample	1st round of passage		2nd round of passage		Result
	Ct value	Log RNA copies/ml	Ct value	Log RNA copies/ml	judgement*
1E5 1# 2 dpi	31.20	5.63	34.91	4.60	Negative
1E5 2# 2 dpi	29.97	5.98	34.07	4.83	Negative
1E5 3# 2 dpi	32.27	5.34	33.9	4.88	Negative
1E5 4# 2 dpi	31.76	5.48	34.2	4.80	Negative
**1E4 1# 2 dpi**	**31.49**	**5.55**	**17.71**	**9.39**	**Positive**
1E4 2# 2 dpi	27.89	6.56	32.39	5.30	Negative
**1E4 4# 2 dpi**	**30.15**	**5.93**	**19.51**	**8.89**	**Positive**
**1E3 4# 2 dpi**	**29.52**	**6.10**	**17.12**	**9.55**	**Positive**

* RNA copies that increased at least 1000-fold was deemed as positive for virus isolation (highlighted in red).

Supplementary Table 2. Genetic diversity of ZIKV in s.c. inoculated guinea pigs

Nucleotide position*	Protein Region	Nucleotide change	Amino acid change
1629	E-253	A→G	None
1707	E-279	A→T	None
1758	E-296	T→C	None
1797	E-309	T→C	None
1815	E-315	C→T	None
1959	E-363	C→T	None
1986	E-372	T→C	None
2004	E-378	T→G	None
2049	E-393	G→A	None
2175	E-435	A→G	None
2178	E-436	C→T	None
2191	E-441	T→C	None
2220	E-450	A→G	None
**2287**	**E-469**	**A→G**	**M→V**
2298	E-476	T→C	None
2304	E-478	C→T	None

Position numbers of matching nucleotides correspond to the ZIKV genome sequence (GenBank number KU820898). The single amino acid substitution was highlighted in red.

Supplementary Table 3. The M469V amino acid substitution of ZIKV in selected samples from the s.c. inoculated guinea pigs

d.p.i	Samples	Number	M469V substitution
3	Serum	1#	Yes
	Serum	2#	Yes
	Tear	1#	Yes
	Tear	2#	Yes
	Saliva	1#	Yes
	Saliva	2#	Yes
	Kidney	1#	No
	Testis	1#	No
6	Serum	3#	Yes
	Serum	4#	Yes
	Tears	3#	Yes
	Saliva	3#	Yes
	Saliva	4#	Yes
	kidney	2#	Yes
	Brain	1#	Yes

Supplementary Table 4. Serum ctokine level in ZIKV-infected cynomolgus monkeys

Items (pg/mL)	i.n. inoculation												i.g. inoculation											
	C0008						C0057						C0077						C0105					
	0 dpi			4 dpi			0 dpi			4 dpi			0 dpi			4 dpi			0 dpi			4 dpi		
	Mean	SD	N	Mean	SD	N	Mean	SD	N	Mean	SD	N	Mean	SD	N	Mean	SD	N	Mean	SD	N	Mean	SD	N
FGF-Basic	26.2	2.1	3	24.7	1.9	3	18.1	1.3	3	15.6	1.7	3	23.2	1.2	3	39.2	15.6	3	22.5	4.0	3	18.0	1.5	3
IL-1b	43.2	4.0	3	29.2	4.0	3	31.8	5.6	3	23.4	1.2	3	8.6	0.0	3	13.6	4.4	3	35.6	7.2	3	25.8	1.3	3
G-CSF	459.6	3.9	3	463.0	8.0	3	697.2	109.4	3	867.9	72.0	3	428.2	6.9	3	506.0	66.5	3	469.9	10.4	3	457.4	12.2	3
IL-10	33.9	0.1	3	35.4	1.3	3	40.0	2.1	3	40.9	1.8	3	50.4	1.4	3	71.1	6.5	3	43.2	3.5	3	42.0	1.1	3
IL-6	25.1	2.4	3	24.4	2.1	3	26.8	6.0	3	30.0	0.3	3	21.1	1.0	3	28.0	7.2	3	26.2	3.3	3	21.8	1.3	3
**IL-12**	**2987.4**	**78.2**	**3**	**5221.5**	**528.4**	**3**	**655.6**	**93.4**	**3**	**804.5**	**34.3**	**3**	**382.0**	**42.2**	**3**	**1318.9**	**151.1**	**3**	3149.2	895.9	3	3036.4	195.7	3
RANTES	9666.4	318.7	3	8934.5	170.3	3	9222.3	411.2	3	10776.0	692.6	3	5008.9	335.6	3	11764.6	388.1	3	9910.8	497.2	3	10712.6	262.7	3
eotaxin	2007.1	277.3	3	1556.5	420.2	3	3175.8	1302.7	3	945.3	113.1	3	344.9	71.1	3	431.8	112.9	3	4437.0	2836.0	3	1065.5	71.3	3
IL-17	56.0	3.4	3	54.4	6.9	3	67.9	15.6	3	67.9	3.2	3	48.8	3.3	3	113.6	43.7	3	177.2	38.1	3	141.4	8.5	3
MIP-1a	117.5	2.4	3	122.8	6.6	3	107.3	4.3	3	105.3	1.4	3	102.2	0.7	3	133.8	30.5	3	137.9	11.0	3	127.6	2.6	3
**GM-CSF**	**96.5**	**10.7**	**3**	**110.7**	**23.0**	**3**	**67.3**	**5.6**	**3**	**100.7**	**4.9**	**3**	62.5	1.8	3	69.6	15.1	3	72.5	6.3	3	68.0	1.7	3
MIP-1b	46.5	3.9	3	61.9	13.1	3	20.3	15.0	3	16.9	20.5	3	28.8	20.5	3	165.1	156.4	3	91.3	39.3	3	53.1	13.8	3
MCP-1	944.7	34.0	3	1079.6	69.0	3	2513.3	170.6	3	1089.7	35.3	3	246.5	25.4	3	747.9	156.1	3	1025.5	74.9	3	779.8	12.5	3
IL-15	99.7	5.1	3	92.4	8.5	3	77.9	8.3	3	68.3	4.7	3	66.5	3.0	3	98.1	38.5	3	128.4	31.4	3	110.3	6.0	3
EGF	566.9	5.4	3	235.2	8.4	3	142.3	8.3	3	295.8	10.1	3	123.3	7.6	3	154.1	58.0	3	431.1	12.8	3	134.3	8.5	3
IL-5	17.9	2.4	3	21.7	5.8	3	8.2	0.5	3	8.4	0.2	3	7.4	0.0	3	8.3	1.1	3	8.2	0.3	3	7.6	0.1	3
HGF	189.0	0.0	3	219.0	47.8	3	203.4	58.4	3	173.2	13.6	3	48.7	20.7	3	329.1	368.4	3	269.6	102.7	3	211.9	22.9	3

VEGF	5.0	0.1	3	5.3	0.5	3	10.5	2.9	3	13.1	1.9	3	2.6	0.6	3	2.7	0.8	3	6.3	0.9	3	5.1	0.3	3
IFN-r	46.0	0.0	3	54.2	2.7	3	42.4	4.1	3	36.2	2.0	3	29.4	6.0	3	272.0	232.8	3	77.8	24.2	3	55.1	4.2	3
**MDC**	1325.8	71.9	3	1760.6	110.4	3	420.9	192.2	3	406.1	50.7	3	**49.0**	**39.4**	**3**	**311.2**	**502.4**	**3**	**1087.5**	**39.9**	**3**	**1489.8**	**119.5**	**3**
I-TAC	395.3	22.7	3	459.9	112.6	3	587.3	243.5	3	143.6	24.7	3	52.6	0.0	3	194.5	200.7	3	254.4	80.4	3	94.5	8.6	3
**MIF**	**7871.6**	**189.0**	**3**	**27587.8**	**1747.2**	**3**	**1963.7**	**44.6**	**3**	**3576.1**	**57.8**	**3**	1162.4	30.6	3	195.4	12.1	3	3176.5	72.7	3	5158.4	100.7	3
IL-Ra	1614.4	46.3	3	6655.9	530.3	3	1735.5	163.8	3	1386.4	43.5	3	459.2	17.8	3	1340.9	436.4	3	1998.0	155.6	3	1305.5	81.4	3
TNF-a	83.7	5.8	3	73.2	7.6	3	76.4	13.1	3	76.6	3.2	3	48.4	0.7	3	62.5	20.8	3	62.9	5.0	3	76.6	2.6	3
IL-2	52.5	0.0	3	61.4	7.7	3	52.5	0.0	3	52.5	0.0	3	52.5	0.0	3	217.6	286.0	3	322.3	95.8	3	123.0	50.8	3
IP-10	23.3	3.7	3	39.4	7.4	3	25.4	3.9	3	22.7	1.1	3	8.3	3.3	3	21.7	23.0	3	40.4	9.2	3	30.8	2.1	3
MIG	72.6	29.4	3	72.6	29.4	3	109.2	41.3	3	25.0	0.0	3	49.4	29.5	3	568.6	544.7	3	379.7	126.5	3	151.5	21.6	3
IL4	141.8	1.4	3	141.0	1.4	3	141.0	1.4	3	138.6	2.8	3	138.6	2.8	3	159.6	35.7	3	145.1	4.9	3	141.4	2.1	3
**IL-8**	**55.4**	**7.8**	**3**	**87.7**	**9.7**	**3**	**334.1**	**16.9**	**3**	**371.0**	**54.6**	**3**	**113.4**	**8.7**	**3**	**128.1**	**8.8**	**3**	**160.5**	**22.0**	**3**	**383.7**	**53.1**	**3**

* Each sample was assayed in triplicate. Data are presented as mean ± SD. The elevated cytokines in at least two animals were highlighted in brown.
